# Precision Medicine in Phaeochromocytoma and Paraganglioma

**DOI:** 10.3390/jpm11111239

**Published:** 2021-11-22

**Authors:** Bettina Winzeler, Benjamin G. Challis, Ruth T. Casey

**Affiliations:** 1 Department of Endocrinology, Diabetology and Metabolism, University Hospital Basel, 4031 Basel, Switzerland; bettina.winzeler@usb.ch; 2 Department of Clinical Research, University of Basel, 4031 Basel, Switzerland; 3 Department of Medical Genetics, Cambridge Biomedical Campus, Cambridge University, Cambridge CB2 0QQ, UK; 4 Department of Endocrinology, Cambridge University Hospital, Cambridge CB2 0QQ, UK; bc340@medschl.cam.ac.uk

**Keywords:** personalized medicine, neuroendocrine tumours, phaeochromocytoma, paraganglioma, molecular clusters

## Abstract

Precision medicine is a term used to describe medical care, which is specifically tailored to an individual patient or disease with the aim of ensuring the best clinical outcome whilst reducing the risk of adverse effects. Phaeochromocytoma and paraganglioma (PPGL) are rare neuroendocrine tumours with uncertain malignant potential. Over recent years, the molecular profiling of PPGLs has increased our understanding of the mechanisms that drive tumorigenesis. A high proportion of PPGLs are hereditary, with non-hereditary tumours commonly harbouring somatic mutations in known susceptibility genes. Through detailed interrogation of genotype-phenotype, correlations PPGLs can be classified into three different subgroups or clusters. Thus, PPGLs serve as an ideal paradigm for developing, testing and implementing precision medicine concepts in the clinic. In this review, we provide an overview of PPGLs and highlight how detailed molecular characterisation of these tumours provides current and future opportunities for precision oncology.

## 1. Introduction

Precision medicine is the provision of individualised healthcare to patients based on specific characteristics that may confer susceptibility to a particular disease and/or response to specific treatment. In this context, interventions can be tailored to maximise benefit whilst reducing exposure to less efficacious or intolerable therapies and unnecessary clinical investigations. The advent of high-throughput DNA sequencing and other molecular profiling technologies has accelerated the discovery of disease causing or ‘driver’ genetic variants as well as other molecular biomarkers associated with disease diagnosis, prognosis and drug-response. In oncology, increased understanding of the genomic, transcriptomic and epigenetic landscapes of multiple solid tumours has paved the way for precision medicine approaches to cancer care. Today, there are select examples whereby physicians have the opportunity to exercise a precision medicine approach to clinically manage and treat patients based on their genotype or the molecular phenotype of their tumour. For example, the use of trastuzumab in HER2-positive breast cancer, vemurafenib for *BRAF*^V600^-mutant melanoma, and olaparib for *BRCA1/2* mutated tumours. Whilst the rationales of targeted therapy in cancer remains clear, the extent to which such an approach will benefit patients on a large-scale remain uncertain and several limitations remain before widespread uptake across multiple tumour types, especially rare tumours, is realised.

Phaeochromocytomas and paragangliomas (PPGLs) are rare neuroendocrine tumours with an annual incidence of 3–8 cases per 1 million per year in the general population [1]. Pheochromocytomas arise from chromaffin tissue from the adrenal medulla, whereas paragangliomas arise from neural crest derivatives of extra-adrenal sympathetic or parasympathetic paraganglia [2]. The management of PPGL patients is challenging for clinicians, as these tumours exhibit marked clinical heterogeneity that includes indolent, slow growing to aggressive and metastatic tumours. Clinical signs and symptoms vary considerably depending on the localisation and size of the tumours and their hormonal activity. PPGL may be non-functional or produce catecholamines such as adrenaline, noradrenaline and dopamine. Catecholamine excess may lead to paroxysmal headache, tachycardia, sweating and hypertension [3,4], and, rarely, to lethal pheochromocytoma crisis [5].

All PPGL have metastatic potential [6] and morbidity and mortality are high in metastatic disease, which is the case in approximately 10% of pheochromocytomas and 45–40% of paragangliomas [7,8,9]. In addition to the clinical complexity, PPGL demonstrate considerable genetic heterogeneity and are considered the most heritable human neoplasms. Historically, patients with PPGL have been stratified according to the anatomic location, developmental origin, primary tumour size, multiplicity, metastatic behaviour or age of first diagnosis. More recently, new approaches including biochemistry, immunohistochemistry and imaging phenotypes have been proposed and used to stratify PPGL patients [10,11]. Moreover, in the area of next generation sequencing (NGS), the possibility of accurate and timely molecular characterisation through germline and somatic testing have accelerated the molecular understanding of PPGL tumourigenesis thereby providing the opportunity for a precision medicine approach to the clinical management of these tumours [12,13].

## 2. Genomic Landscape of PPGL

Knowledge of the genetic status of PPGL patients is key for adequate diagnosis, treatment and surveillance of affected patients and their families, and has shown to positively impact on management and patient outcome [14]. Consequently, current guidelines recommend germline genetic testing in every patient with PPGL, with NGS as the gold standard for routine diagnosis [15,16,17]. One third of patients are affected by a germline mutation in one of the currently known susceptibility genes [11]. Germline mutations in the *RET, VHL* and *NF1* genes predispose to phaeochromocytoma in the context of inherited tumour syndromes, including neurofibromatosis, multiple endocrine neoplasia type 2 (MEN 2) and Von Hippel Lindau syndrome (VHL) [18,19]. Non-syndromic, hereditary PPGLs are mostly due to germline mutations in the succinate dehydrogenase genes (*SDHx* group) while other familial PPGL are caused by *TMEM127* or *MAX* mutations. Newer syndromic PPGL genes include *FH* (Hereditary Leiomyomatosis and Renal Cell Cancer syndrome), *EPAS1* (mainly as a case of germline mosaicism, although *EPAS1* mutations are generally more common as somatic mutations) and *EGLN1* (associated with congenital polycythaemia). In single cases, additional PPGL susceptibility genes have been described and are implicated in mitochondrial metabolism (*MDH2, GOT2, SLC25A11, DLST*), mitogen-activated protein kinase (MAPK) signaling pathways (*MET, MERTK*) or DNA methylation (*H3F3A, DNMT3A, KIF1Bbeta*) [10,20,21].

In addition to germline genetic testing, identification of somatic mutations by sequencing tumour DNA is increasingly undertaken in both research and clinical settings. Testing can be applied to paraffin embedded tissues as well as fresh frozen samples [22] and helps to further improve knowledge of tumour biology by rapidly identifying driver mutations that may be amenable to personalised treatment strategies or inform surveillance strategies.

**Figure 1:**
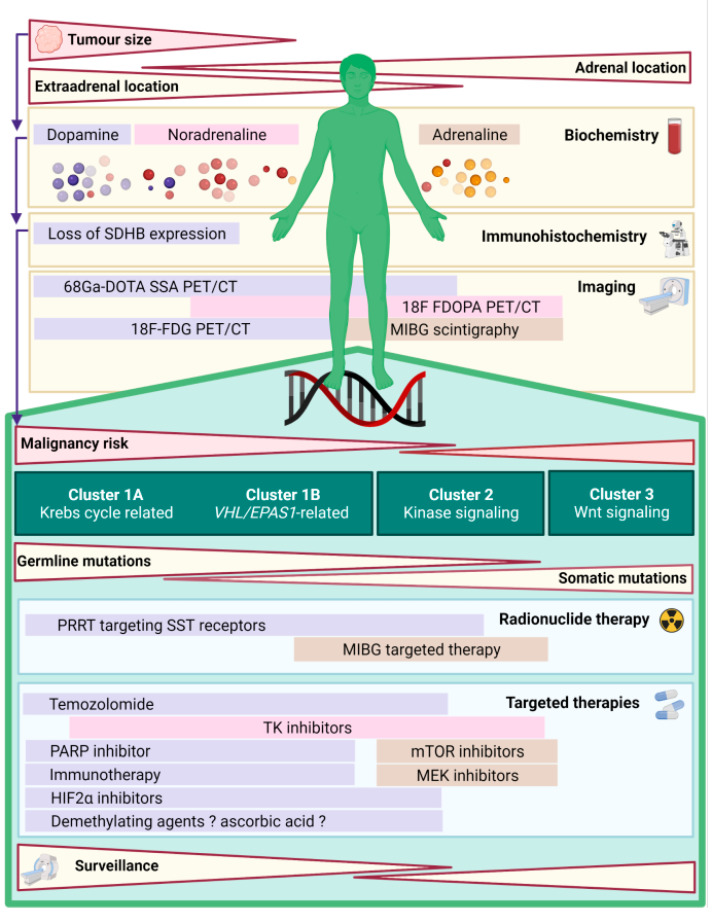
Provides an overview of how molecular classification can guide a personalized approach to PPGL patients. PARP: poly-(ADP)-ribose polymerase, mTOR: mechanistic Target of Rapamycin, MEK: mitogen-activated protein kinase kinase (MAP2K), TK: tyrosine kinase, MIBG: metaiodobenzylguanidine, PRRT: peptide receptor radionuclide therapy, SST: somatostatin.

Up to 40% of cases without an established germline mutation may harbor a somatic mutation in one of the PPGL susceptibility genes such as *VHL, RET, EPAS1, SDHB, NF1* or in genes implicated in oncogenesis (*HRAS, TP53, CDKN2A, FGFR1*) [22,23,24]. The number of genetically determined tumours with a specific driver mutation rises, therefore, to approximately 70% [25]. Based on their underlying driver mutation, PPGL can be divided in three different molecular clusters [13] that reflect different mechanisms of tumourigenesis and are associated with distinct biochemical, radiological and clinical phenotypes, see Table 1 and Figure 1.

## 3. Mechanisms of Tumourigenesis in PPGL and Molecular Clusters

The first cluster is characterised by a pseudohypoxic signature and accelerated angiogenesis promoted by abnormal stabilization of hypoxia-inducible factor (HIF) alpha transcription [26]. Pseudohypoxic PPGL can further be subdivided in Krebs cycle-related (Cluster 1A) and *VHL/EPAS1* related (Cluster 2B) tumorigenesis. Cluster 1A Krebs cycle-related tumours are secondary to mutations in *SDHx*, *FH, MDH2*, *GOT2*, *SLC25A11*, *DLST* or *IDH1/2*. The *SDHx* genes (*SDHA*, *SDHB*, *SDHC*, *SDHC*, *SDHAF2*) are the most commonly affected genes and encode different units and assembly proteins of the mitochondrial enzyme succinate dehydrogenase (SDH) [18] which is implicated in the Krebs cycle and oxidizes succinate to fumarate. The disruption of Krebs cycle enzymes leads to accumulation of the oncometabolites succinate (*SDHx*), fumarate (*FH*) or 2-hydroxyglutarate (*IDH*) that drive tumorigenesis by DNA hypermethylation, inactivation of tumour suppressor genes and HIF alpha stabilisation [27].

Cluster 2B *VHL/EPAS1* related PPGL are due to mutations in the *VHL*, *EPAS1*, *EGLN1/2* and *IRP1* genes and are characterised by the key pathogenic mechanism of stabilisation and accumulation of HIF alpha proteins. The pseudo-hypoxic state of Cluster 1 tumours results in increased angiogenesis (e.g., vascular endothelial growth factor (VEGF) transcription), cellular proliferation and reduced apoptosis [28,29].

The second cluster is characterised by an upregulation of kinase signaling pathways involving the MAPK pathway and the mechanistic Target of Rapamycin (mTOR) pathway leading to enhanced cell growth and cell survival. These tumours arise as a result of gain-of-function-mutations in *RET* or mutations in genes such as *NF1*, *TMEM127*, *MAX*, *MET*, *MERTK* and *FGFR1* and *HRAS* (both somatic) [13].

Finally, the third cluster comprises genes leading to activation of the Wnt/beta-catenin pathway resulting in increased angiogenesis, cell proliferation and invasion. Perturbations of this pathway are less explored and were exclusively described in sporadic PPGL with somatic variants in *CSDE1* and *MAML3* fusion genes [13].

Generally, Cluster 1 genes are more commonly affected at the germline level, while Cluster 2 genes mutations are observed both at the germline and somatic level [13,23].

## 4. Clinical Phenotype According to Molecular Clusters

Besides the presence of typical syndromic features or a specific family history, a range of clinical, biochemical, histopathological or imaging characteristics may inform prediction about a genetic cause and the underlying molecular cluster, and therefore, may guide a personalized management from an early stage. Young age at presentation suggests the presence of a germline pathogenic variant. Extra-adrenal localisation and large tumour size at diagnosis may predict a germline *SDHx* gene mutation while adrenal localization may point to a Cluster 2 mutation [30]. Episodic symptoms or a higher symptom score (e.g., tremor, pallor, anxiety) are suggestive of an adrenaline producing (mostly Cluster 2 related) PPGL. Cluster 1 and possibly Cluster 3 PPGLs show a more aggressive phenotype (with the highest metastatic risk for *SDHB* mutations) compared with Cluster 2 tumours [31]. According to a recent meta-analysis, the metastatic risk for Clusters 1, 2, and 3 PPGLs are 24% (40.5% for Cluster 1A), 4.1% and 11.4%, respectively [31]. In patients with metastatic PPGL the probability of an underlying mutation in Cluster 1 genes are as high as 60% [32,33,34].

## 5. Biochemistry

The recommended test to establish the biochemical phenotype of PPGLs is the measurement of the respective O-methylated metabolites of adrenaline, noradrenaline and dopamine (metanephrine, normetanephrine and 3-methoxytyramine) ideally in plasma via liquid chromatography-tandem mass spectrometry (LH-MS) or urine [17]. Blood for plasma metanephrines should be drawn in a supine position and the patient should ideally be recumbent for at least 30 min before sampling. The sensitivity of plasma free metanephrines (as well as of 24-h urine fractionated metanephrines) is very high (96–100%) and a negative test result virtually excludes PPGL. The specificity or plasma free metanephrines is lower (around 89%) due to medication or physiological stress that may interfere with the measurement and sampling should be repeated and confirmed by another test modality (24-h urine fractionated metanephrines or clonidine test [17,35]).

From the biochemical phenotype conclusions can be drawn about molecular tumour characteristics and cell differentiation. A noradrenergic phenotype (with increased normetanephrines levels) points to a tumour lacking the enzyme phenylethanolamine N-methyl transferase, which converts noradrenalin to adrenalin [36]. A dopaminergic phenotype (with increased 3-methoxytyramine levels) is suggestive of a deficiency in the enzyme dopamine-beta-hydroxylase, which converts dopamine to noradrenaline [37]. PPGLs of the pseudohypoxia group (Cluster 1) and especially *SDHx* mutation carriers are associated with a noradrenergic or dopaminergic phenotype reflecting the poor differentiation of paraganglia cells and reduced converting enzyme expression [38]. Some *SDHx* mutation carriers also lack the enzyme tyrosine hydroxylase (catecholamine synthesis) and are, therefore, characterised by a non-functional phenotype. Conversely, Cluster 2 tumours typically show a mixed or predominately adrenergic secretory phenotype as a consequence of more mature cell differentiation and increased expression of phenylethanolamine N-methyl transferase.

Stratification according to the hormonal activity of the tumour helps to decide whether or not patients need pre-operative treatment with alpha-blockade but may also guide sequential genetic testing. Longitudinal comparison of metabolites gives information about disease control or progression [39]. In non-functional tumours surveillance is based on repeated cross-sectional imaging and the measurement of the biomarker chromogranin A [40,41,42]. Finally, highly elevated 3-methoxytyramine levels are associated with a more aggressive behaviour of the tumour and may suggest metastatic disease (an exception to this rule is *SDHx*-mutated, non-metastatic head and neck paraganglioma that may show elevation of 3-methoxytyramine) [43]. Such patients should undergo pre-operative staging, ideally with functional radionuclide imaging (see below), and need increased surveillance.

## 6. Immunohistochemistry

In general, neuroendocrine tumours are characterised by positive immunohistochemistry for chromogranin A, synaptophysin, and S100 [25]. In PPGL, a relevant metabolic biomarker is SDHB immunohistochemistry which allows for the identification of patients with mutations in the *SDHx* tumour suppressor genes [44,45,46]. Biallelic inactivation (e.g., germline variant and “second hit” at the somatic level) in the *SDHx* genes disrupts the SDH enzyme complex and the anchor SDHB protein [45]. Loss of SDHB protein expression is seen in PPGLs with mutations in any of the *SDH* genes (*SDHA*, *SDHB*, *SDHC*, *SDHD*, *SDHAF2*) or with somatic hypermethylation of the *SDHC* promoter region [47]. Conversely, negative immunohistochemistry for SDHB and SDHA is only seen in tumours with SDHA mutations. The sensitivity and specificity of SDHB immunohistochemistry are around 94% and 85%, respectively. In *SDHD* mutated tumours, the SDHB immunohistiochemistry may be misleading, as sometimes interpreted as immunopositive. Conversely, immunonegative results in some *VHL* and *NF1* mutated tumours compromise specificity of SDHB immunohistochemistry [47].

SDHB immunohistochemistry allows a targeted approach to genetic testing and has also a value in the pathogenicity assessment of genetic variants of unknown significance. Moreover, SDHB immunohistochemistry in PPGL is associated with metastasis and poor outcome and is, therefore, a marker of malignancy.

Immunohistochemistry may also have a role in interpreting variants in *FH* by assessing loss of expression of the fumarate hydratase protein or in screening for *MAX*- and *VHL*-related PPGLs by immunohistochemical staining for MAX and carbonic anhydrase 9 expression, respectively [48,49].

In analogy to immunohistochemistry, the measurement of metabolites such as succinate, fumarate, malate or 2-hydroxyglutarate (2HG) provides also a screening tool to identify underlying driver mutations in PPGL or to assess functionality of variants of unknown significance. PPGL with high succinate:fumarate ratios point to *SDHx* mutations, while a high fumarate:malate ratio suggests a FH mutation. High levels of 2HG (combined with a high D- to L-enantiomers ratio of 2HG) may identify patients at risk for IDH1/2 mutations [46,50].

## 7. The Influence of Genotype on Molecular Imaging Modality Selection in PPGL

Molecular imaging techniques have an important role in the management of PPGL in clinical practice and may be used for: (i) PPGL staging, (ii) localisation of an occult tumour(s) and (iii) theranostic applications to determine the response to radionuclide therapies.

Molecular imaging tracers specific for PPGL can be sub-classified into three main groups based on their target ligand and include: (i) catecholamine storage and synthesis; [123I-metaiodobenzylguanidine, 18F-fluorodopamine (18F-FDA) and 18F-fluorodihydroxyphenylalanine (18F-FDOPA)], (ii) glucose metabolism [18F-fluorodeoxyglucose (18F-FDG)] and (iii) somatostatin receptor [111indium–pentetreotide and gallium-68 DOTA-conjugated peptide (68Ga-DOTATATE)]. The selection of the most appropriate tracer is by patient genotype and the affect that genotype has on tumour biology, anatomical location and secretory pattern, all of which influence the expression of receptors targeted by molecular imaging tracers, thereby giving rise to a so-called molecular imaging phenotype [51].

### 7.1. Radiotracers Targeting Ligands Involved in Catecholamine Synthesis and Storage

Commercially available metaiodobenzylguanidine (MIBG) is radiolabelled with ^123^I (Iobenguane) or ^131^I and binds to the noradrenaline transporter (NET) and is stored within catecholamine secreting tumours in neurosecretory granules via vesicular monoamine transporters (VMATs). However, the sensitivity of 123/131I-MIBG scintigraphy is reduced in de-differentiated tumours where expression of the NET or VMATs are reduced or absent, therefore leading to a false-negative results. Furthermore, mutations in ‘Cluster 1’ genes, specifically the *SDHx* genes and *VHL*, are associated with reduced expression of the NET transporter, thereby affecting the sensitivity of 123/131I-MIBG scintigraphy for the detection of primary or metastatic PPGL in patients harbouring these gene mutations. The recognition that false negative results with 123/131I-MIBG scintigraphy may be more common in patients with metastatic PPGL or ‘Cluster 1’ gene mutations has informed current consensus guidelines [15]. It is now recommended that 123/131I-MIBG scintigraphy is reserved for those cases being investigated for suitability of treatment with 123/131I-MIBG radionuclide therapy, particularly in those patients with suspected *SDHx* mutations [15].

The 18F-FDA also binds to the NET transporter and therefore has the same sensitivity issues as 123/131I-MIBG for de-differentiated tumours or patients with ‘Cluster 1’ gene mutations. Finally, the imaging tracer 18F-fluorodopa (FDOPA) binds to the neutral amino acid transporter system L. The sensitivity of 18F-FDOPA PET-CT is also reduced in patients with ‘Cluster 1’ gene mutations, specifically the *SDHx* genes, and this has been attributed to the impaired catecholamine synthesis pathway and truncated citric acid cycle in SDH-deficient tumours. Therefore, patients with ‘Cluster 1’ gene mutations have a specific molecular imaging phenotype that includes reduced or absent avidity of tracers involved in catecholamine synthesis or storage. The opposite molecular imaging phenotype is noted for patients with ‘Cluster 2 gene’ mutations in whom the catecholamine synthesis pathway is often upregulated and therefore tracers binding to NET and VMATs are often more sensitive in patients with ‘Cluster 2’ gene mutations. Therefore, 123/131I-MIBG scintigraphy may be considered as the first-line imaging modality in patients with Cluster 2 gene mutations or sporadic tumours [52].

### 7.2. Tracers Targeting Glucose Metabolism

Imaging with 18F-fluorodeoxyglucose positron emission tomography (18F-FDG PET) is employed in clinical practice to probe the increased glucose use that occurs in many metabolically active tumours and cancers. The sensitivity of 18F-FDG PET-CT in the imaging of PPGL is also influenced by genotype. ‘Cluster 1’ tumours exhibit increased glycolysis due to pseudohypoxia leading to upregulation of glycolytic enzymes and glucose transporters and therefore have higher standard uptake values (SUVs) of 18F-FDG compared to sporadic or ‘Cluster 2’ tumours although FDG avidity in these tumours does not correlate with malignant potential [51].

### 7.3. Tracers Targeting Somatostatin Receptors

The 68Ga-labeled DOTA peptides such as DOTA(0)-Tyr(3)-octreotate (DOTATATE), DOTA(0)-Phe(1)-Tyr(3)-octreotide (DOTATOC), and DOTA(1)-Nal(3)-octreotide (DOTANOC) bind to somatostatin receptors (specifically somatostatin receptor 2) and combined with PET/CT are now the favoured molecular imaging modality for PPGL, largely replacing Technitium (Tc)-99m and Indium (In)-111-labeled Octreotide SPECT/CT [52]. The 68Ga-DOTATATE PET-CT is now recommended as the imaging modality of choice for patients with *SDHx*-mutated PPGL or sporadic or metastatic PPGL and can also predict the efficacy of peptide receptor radionuclide therapy with 177Lu-DOTATATE [52]. The 68Ga-DOTATATE can also help to differentiate between a PPGL and other tumour, such as a gastrointestinal stromal tumour (GIST) in patients with *SDHx* mutations, who are at risk of both tumour types. In such cases, 68Ga-DOTATATE may be preferred over 18F-FDG, which is frequently positive in both tumour types [53].

Finally, as 68Ga-DOTATATE is also taken up by the background normal adrenal gland, sensitivity of this tracer is reduced for patients at risk of small or bilateral phaeochromocytomas such as patients with ‘Cluster 2’ gene mutations. In this instance, 18F-DOPA PET-CT is preferred because of the superior tumour to background uptake of this tracer [54].

## 8. In Vivo Detection of Oncometabolites in PPGL

Tumorigenesis caused by Kreb’s cycle gene defects are driven by accumulation of metabolites referred to as ‘oncometablites’, such as succinate in patients with *SDHx* mutations or fumarate in patients with *FH* mutations. The early detection of oncometabolite accumulation in tumours in vivo can facilitate early personalised management of patients with PPGL. Molecular imaging facilitates the characterisation of tumours at a molecular level and despite advances and improved sensitivity of molecular imaging tracer the ability to measure individual metabolites within a tumour is beyond the scope of these tests. However, techniques such as proton magnetic resonance spectroscopy (^1^H-MRS) can detect metabolite accumulation in PPGL in vivo [55,56]. Our group has previously demonstrated that ^1^H-MRS can detect succinate and fumarate in tumours in vivo and may be used for the early detection of metabolically driven tumours as well as serving as an early efficacy biomarker to monitor treatment response [55,57]. Thus, the detection of metabolite accumulation in tumours in vivo using ^1^H-MRS, offers the opportunity to apply a non-invasive technique to stratify suitable patients with PPGL for specific targeted therapies and may serve as a pre-selection tool for recruitment into clinical trials.

## 9. Precision Medicine for the Treatment of PPGL

### 9.1. Surgery

The first-line treatment option for a primary PPGL or localised disease is surgery. The genotype, if known pre-operatively, may influence the surgical strategy as it provides information on the risk of synchronous or bilateral tumours and metastatic potential. For patients with a *SDHx* gene mutation and a large para-aortic paraganglioma, for example, an open approach may be favoured over a laparoscopic approach to facilitate clear resection margins and lymph node dissection. Whereas patients at risk of bilateral phaeochromocytoma due to mutations in the *VHL*, *RET* or *NF1* genes may be considered for a cortical sparing adrenal surgery to mitigate against the need for life-long glucocorticoid and mineralocorticoid replacement therapy [58]. Although patients with *SDHx* mutations may also develop bilateral phaeochromocytoma, a total adrenalectomy is favoured over a cortical sparing approach because of the higher malignant potential of *SDHx* mutated tumours [59].

### 9.2. Radionuclide Therapies

Radiopharamaceutical therapies for patients with metastatic, inoperable PPGL include ^131^I-MIBG, ^90^Y and ^177^Lu-DOTATATE. The first step in evaluating the potential efficacy of radionuclide therapies is to consider imaging with 68Ga-DOTATATE PET/CT or 123/131I-MIBG scintigraphy. A radionuclide therapy should only be selected if there is evidence of tumoral uptake of the relevant tracer in the tumors demonstrated on either cross-sectional imaging or 18F-FDG-PET/CT [60]. Therefore, genetics can influence the selection of these radiopharmaceutical therapies because of the above-mentioned affect that genotype has on the expression of radiopharmaceutical ligands such as NET in patients with ‘cluster 1’ gene mutations. To date, there are no phase III studies that have investigated the efficacy of ^131^I-MIBG versus ^90^Y- or ^177^Lu-DOTATATE in patients with metastatic PPGL [60].

### 9.3. Cytotoxic Chemotherapy

Combination chemotherapy consisting of cyclophosphamide, vincristine, and dacarbazine (CVD) is the standard cytotoxic chemotherapy regime for patients with metastatic PPGL. Treatment with CVD has demonstrated partial response rates (including tumour volume and catecholamine burden) of up to 40% in unselected cohorts of patients with metastatic PPGL [61]. Anecdotal case reports have suggested that response rates to CVD chemotherapy may be superior in patients with *SDHB* gene mutation [62], and a more recent retrospective study also found that median progression free survival after CVD therapy was superior in patients with *SDHB* gene mutations and metastatic PPGL compared to those without *SDHB* gene mutations [63].

### 9.4. Alkylating Agents

Temozolomide is an alkylating agent originally developed as an oral alternative to intravenous dacarbazine. The expression of O(6)-methylguanine-DNA methyltransferase (MGMT) within cancer cells allows the cell to recover from the DNA damaging effects of Temozolomide, enabling the tumour to become resistant to therapeutic use of such agents. A large retrospective study demonstrated therapeutic efficacy of temozolomide in patients with metastatic PPGL and partial responses were specifically observed in patients with *SDHB* gene mutations [64]. This reported efficacy of temozolomide in *SDHB* mutated PPGL has been attributed to epigenetic silencing of the *MGMT* gene in the *SDHB* mutated tumours [64].

### 9.5. Tyrosine Kinase Inhibitors

PPGL are vascular tumours and studies have shown that angiogenic factors like VGEF, are preferentially expressed in PPGL [65]. Furthermore, the pseudohypoxic phenotype of ‘Cluster 1’ mutated PPGL promotes upregulation of vascular growth factors and, therefore, tyrosine kinase inhibitors (TKIs) have a potential role in the management of metastatic PPGL. Data from a recent phase II study investigating the role of sunitinib in the treatment of metastatic PPGL, has suggested that patients with germline mutations in *RET* or *SDHx* may benefit most [66].

## 10. Emerging Targeted Therapies for PPGL

### 10.1. Cluster 1 PPGL

#### 10.1.1. PARP Inhibitors

Recent studies have demonstrated that accumulation of oncometabolites such as succinate, fumarate or 2-hydroxyglutarate (2-HG) may render tumours susceptible to synthetic-lethal targeting with poly-(ADP)-ribose polymerase (PARP) inhibitors [67,68]. Oncometabolite accumulation inhibits α –ketoglutarate-dependent dioxygenases and the subsequent inhibition of two key lysine demethylases; KDM4A and KDM4B, which ultimately dysregulates DNA homologous repair [68]. A currently recruiting phase II clinical trial is investigating whether the combination of the PARP inhibitor, olaparib, and temozolomide is more efficacious that temozolomide monotherapy in patients with metastatic PPGL (NCT04394858). If proven effective, there may be a role in the future for in vivo or ex vivo analysis for oncometabolite accumulation in order to stratify those patients predicted to be more responsive to PARP inhibition.

#### 10.1.2. Immunotherapy

The pseudohypoxic tumour environment in ‘Cluster 1’ mutated PPGL may facilitate immune evasion through inactivation of cytotoxic T-cell lymphocytes and stimulation of immune-suppressive monocytes [69] and this finding has prompted interest in the role of immune modulating drugs for PPGL. Two phase II studies investigating the efficacy of immunotherapies such as pembrolizumab (NCT02721732) and Nivolumab plus ipilimumab (NCT02834013) in rare cancers including PPGL, are currently recruiting.

#### 10.1.3. Demethylating Agents

Demethylating agents may have therapeutic benefit in Cluster 1 mutated PPGL because of the hypermethylation phenotype promoted by mutations in Kreb’s cycle genes [70]. The role of hypomethylating agents prompted interest in new second-generation demethylating agents such as guadecitabine (SGI-110), which has superior bioavailability compared with its parent drug decitabine [71], and is licensed for use in solid tumours. However, a recent phase 2 clinical trial investigating Guadecitabine (SGI-110) monotherapy (NCT03165721) in patients with *SDHx* or *FH* mutated tumours was terminated because of low accrual. Of the nine subjects enrolled including seven with SDH-deficient GIST, one with SDH-deficient paraganglioma and one *FH*-mutated RCC, no complete or partial responses were observed [72].

#### 10.1.4. HIF2α Antagonists

Hypoxia-inducible factor 2 *α* (HIF-2 *α*) is strikingly upregulated in ‘Cluster 1’ mutated PPGL and has sparked great interest in applying inhibitors of HIF as anticancer therapies. A pre-clinical study demonstrated efficacy of a small molecule HIF-2αinhibitor (PT2399) in mouse models of primary and metastatic pVHL defective clear cell renal cell carcinoma [73]. More recently, two phase II clinical trials evaluating the role of a HIF2α (PT2385) in VHL associated RCC (NCT03108066) and the effectiveness of Belzutifan/MK-6482 for the treatment of advanced PPGL and pancreatic neuroendocrine tumours, respectively, are currently ongoing (NCT04924075). These studies will hopefully inform the therapeutic utility of HIF2α inhibitors for advanced PPGL as well as tumours harbouring *EPAS1*, *VHL*, *SDHX*, *FH* and other somatic and germline ‘Cluster 1’ gene mutations.

#### 10.1.5. Targeting Metabolic Reprogramming and Redox Imbalance

The metabolic vulnerability of tumours harbouring citric acid cycle gene mutations provides an opportunity for therapeutic targeting. Ex vivo metabolomics analysis on tumour samples performed by this group has demonstrated that the metabolomics fingerprint of *SDHx* mutated PPGL includes a significant reduction in metabolites such as aspartate (unpublished data). Aspartate is essential for DNA synthesis and cellular proliferation and in vitro studies have suggested that SDH deficient tumours overcome the deficiency in aspartate by up-regulating the enzyme pyruvate carboxylase [74]. A small molecule inhibitor of oxidative phosphorylation (IACS-010759) is currently being evaluated for safety in phase 1 studies (NCT02882321 and NCT03291938). This molecule arrests proliferation by inhibiting aspartate production, thereby impairing nucleotide biosynthesis and therefore may be a promising therapy for SDH deficient tumours.

Finally, impaired oxygen sensing pathways in ‘Cluster 1’ mutated PPGL leads to accumulation of oxygen free radicals and iron. In vitro studies have suggested that pharmacological does of ascorbic acid can induce an overload of reactive oxygen species in in *SDHB* knockdown cells, promoting apoptosis [75] and this has prompted interest in the therapeutic role of ascorbic acid alone or in combination with other therapies for ‘Cluster 1’ mutated PPGL.

### 10.2. Cluster 2 Mutated PPGL

#### New Kinase Inhibitors

Upregulation of signalling pathways such as the Ras/Raf/Erk or PI3K/Akt/mTOR pathways is most commonly seen in PPGL with Cluster 2 germline gene mutations e.g., *NF1*, *RET*, *TMEM127* or somatic mutations in *NF1* or *HRAS*. A phase II study investigating the benefit of the mTOR inhibitor everolimus in metastatic neuroendocrine tumours and PPGL demonstrated modest efficacy in the small number of metastatic PPGL patients enrolled [76]. Recently inhibition of MAPK signaling using mitogen-activated protein kinase kinase (MEK) inhibitors has demonstrated efficacy in the treatment of malignant peripheral nerve sheath tumours harboring *NF1* mutations in children [77] and raises the possibility that MEK inhibitors may have a therapeutic role in molecular subsets of metastatic PPGL such as those with ‘Cluster 2’ gene mutations (Figure 1).

## 11. Future Considerations for Precision Medicine in PPGL

With technological advances contributing towards an improved understanding of the molecular basis for PPGLs for a large proportion of patients, it seems that we have entered the era of precision healthcare for individuals with PPGL. Indeed, this notion is reflected in recent published guidance, which recommends that all patients with primary PPGL or metastatic PPGL have clinical germline genetic testing [13,14,16]. Such recommendation implies that a genetic diagnosis will inform personalised medicine strategies, which will guide individualised clinical management and, ultimately, improve patient outcomes. However, several challenges exist which may limit the widespread incorporation of precision medicine in routine clinical care of patients with PPGL.

In order for precision medicine to have widespread impact, genomic profiling technologies used to stratify patients need to be affordable, widely accessible and supported by the appropriate infrastructure. The requirement of tumour tissue for next generation sequencing or gene expression profiling necessitates that tumour specimens obtained through biopsy or surgical resection are sampled, collected and stored correctly to mitigate against technical failures due to poor sample quality. In many instances, due to a reluctance or inability to undertake repeated tumour biopsies because of the associated risk of catecholamine excess and need for alpha blockade, archival tissue is analysed in attempt to molecularly stratify tumours and ‘match’ their molecular signatures with targeted therapies. However, the genomic landscape of archival tissue may not accurately reflect tumour evolution and intra-tumoral heterogeneity in recurrent, progressive or metastatic disease. For some cancers, a ‘liquid biopsy’ containing circulating tumour DNA (ctDNA) has been proposed as an alternative to repeat tumour biopsies and may provide ‘real-time’ information regarding genomic alterations within a tumour and identify key driver mutations [78]. Recently, the diagnostic utility of the NETest, a NET-specific 51-marker gene blood assay, was investigated in 81 subjects with PPGL [79]. The investigators found that the NETest was able to diagnose PPGLs with 100% efficacy and differentiate Cluster 2 from Cluster 1 tumours thereby providing proof of concept evidence that ‘liquid biopsies’ may have clinical utility in the management of PPGLs. When biomarkers, such as ctDNA, or other molecular diagnostic tests, are used to stratify patients into smaller sub-groups and accompany a targeted therapy, they may be referred to as a companion diagnostic. A companion diagnostic is a regulatory approved test that has been validated, both analytically and clinically with the therapy. Companion diagnostics are often co-developed alongside a new targeted therapy in a lengthy process and usually at a considerable cost. Thus, when developed for smaller patient sub-groups with rare cancers, such as PPGLs, targeted therapies with accompanying companion diagnostics may be prohibitively expensive for some healthcare systems.

To justify the rationale and cost of precision medicine approaches to the management of PPGLs to healthcare providers, patients, regulators and payers, it is crucial that such strategies are backed by evidence that demonstrate patient benefit. That consensus guidance recommends genetic testing for the majority of patients with PPGL suggests that confirmation and awareness of a genetic diagnosis confers greater benefit to patients compared with those unaware of their genetic status. Indeed, Buffet et al., found that PPGL patients who were informed of their positive genetic status for *SDHx* and *VHL* mutations underwent more examinations, were less likely to be lost to follow-up and had an improved 5-year survival rate in the presence of metachronous metastases when compared to a historic cohort of subjects who were not aware of their genetic diagnosis [14]. Thus, knowledge of a genetic diagnosis influences how patients engage with healthcare providers which equates to improved clinical outcomes. Additional clinical studies which prospectively follow individuals with mutations in PPGL susceptibility genes and records compliance with surveillance and corresponding clinical outcomes are warranted.

The most desired outcome of precision oncology is the ability to match the genomic profile of a tumour with a well-tolerated and efficacious targeted therapy. However, such a strategy may be difficult to implement due to a lack of available medicines that ‘match’ the molecular landscape of a tumour. Moreover, if a candidate therapy is available for a specific patient subgroup generating the required clinical trial evidence to support its use in the clinic can be challenging, especially for rare tumours. The traditional randomised placebo-controlled clinical trial may not be the optimal clinical trial design for studying the safety and efficacy of new investigational therapies in rare diseases. This may be due to a number of factors, which includes a small number of recruitable patients resulting in long and expensive clinical trials that may be underpowered. However, as new precision medicine-based therapies are developed, clinical trial design has also evolved, especially in oncology, to accommodate the need for innovative approaches for studying new treatments.

The ‘basket’ trial design is intended to study a single investigational drug across a number of tumours (tumour agnostic) that share a common feature (for example, molecular profile). Therefore, in a basket design the study population is enriched by including only those participants with biomarkers that render them most likely to respond to the intervention. In 2018, pembrolizumab was the first drug to receive FDA (Food and Drug Administration) approval for a tissue agnostic indication based on demonstrated efficacy across a number of solid tumours characterised by mismatch repair deficiency or high microsatellite instability. A similar approach could be adopted for the study of new treatments for PPGL and related tumours. For example, it is well-established that SDH deficiency not only predisposes individuals to developing PPGL but other tumours, including gastrointestinal stromal tumours (GIST) and renal cell carcinoma [80]. Thus, adoption of the ‘basket’ design across the spectrum of *SDHx* mutated tumours may be an effective way of studying new or repurposed drugs in this patient group. Another example of clinical trial innovation that supports the development of therapies targeting rare diseases is the use of real-world evidence (RWE). Here, advanced data analytics and RWE can be leveraged to create an observational placebo arm to support a clinical trial. Such an approach minimises the number of people required for a control arm and provides opportunity for more patients to potentially benefit from a new targeted treatment.

Finally, if precision medicine approaches to PPGL and other cancers are to become commonplace in the clinical setting, it is imperative that endocrinologists and oncologists are equipped with the knowledge required to exercise this practice. Physicians caring for these patients will need to have familiarity with genomics and genetics and the interpretation of genetic test results. They will need to possess the qualification required to accurately communicate the findings of genomic tests, and their implications, to their patients and their families. Physicians will need to understand the fundamentals of molecular technologies including their advantages and disadvantages, and maintain their knowledge of the ever evolving scientific and therapeutic landscape.

## Figures and Tables

**Table 1:** Genes and hallmarks of tumourigenesis according to molecular clusters.

	Cluster 1	Cluster 2	Cluster 3
Genes	Cluster 1A: *SDHx*, *FH*, *MDH2*, *IDH1/2*, *SLC25A11*, *DLST*, *GOT2*, *DNMT3A*, *EGLN1*	*RET*, *NF1*, *TMEM127*, *MAX*, *HRAS*, *KRAS*, *FGFR1*, *NGRF*, *KIF1B*, *BRAF*, *MET*, *MERTK*	*MAML3*, *CSDE1*
	Cluster 1B: *VHL*, *EPAS1*, *EGLN1/2 IRP1*		
Hallmarks of tumourigenesis	Pseudohypoxia	Increased Cell proliferation	Activated Wnt/β-catenine pathway
	Angiogenesis		
	DNA and Histone methylation	Increased cell survival	
	Metabolic reprogramming		
